# Dietary patterns and gut microbial clusters jointly stratify frailty in community-dwelling older Japanese adults: a cross-sectional study in the Kyotango longevity area

**DOI:** 10.1016/j.jnha.2026.100946

**Published:** 2026-08-12

**Authors:** Yuji Naito, Takeshi Yasuda, Hiroaki Kitae, Katsura Mizushima, Norihiro Ouchi, Atsuo Adachi, Tadaaki Kamitani, Jin Narumoto, Tomoya Kitani, Satoaki Matoba, Ryo Inoue, Tomohisa Takagi

**Affiliations:** a Department of Human Immunology and Nutrition Science, Graduate School of Medical Science, Kyoto Prefectural University of Medicine, Kyoto, 602-8566, Japan; b Molecular Gastroenterology and Hepatology, Graduate School of Medical Science, Kyoto Prefectural University of Medicine, Kyoto 602-8566, Japan; c Department of Internal Medicine, Kyotango Municipal Yasaka Hospital, Kyoto, 627-0111, Japan; d Department of Psychiatry, Graduate School of Medical Science, Kyoto Prefectural University of Medicine, Kyoto, 602-8566, Japan; e Department of Cardiovascular Medicine, Graduate School of Medical Science, Kyoto Prefectural University of Medicine, Kyoto, 602-8566, Japan; f Department of Longevity and Community Epidemiology, Graduate School of Medical Science, Kyoto Prefectural University of Medicine, Kyoto, 602-8566, Japan; g Laboratory of Animal Science, Department of Applied Biological Sciences, Faculty of Agriculture, Setsunan University, Osaka 573-0101, Japan

**Keywords:** Frailty, Dietary patterns, Gut microbiota, Short-Chain fatty acids, Older adults

## Abstract

**Background:**

Habitual diet and the gut microbiota each influence frailty in older adults, but whether they contribute jointly and whether the fiber–frailty association is statistically mediated by gut microbial composition remain poorly characterized in Asian populations.

**Objective:**

To quantify the joint contribution of dietary patterns and gut microbial clusters to frailty and to test whether a butyrate-producer-rich microbial axis statistically mediates the cross-sectional fiber–frailty association in older Japanese adults.

**Methods:**

In 785 community-dwelling participants aged ≥65 years from Kyotango, a Japanese longevity area, BDHQ-derived intakes (27 food groups) and 16S rRNA gene amplicon sequencing of fecal samples (47 CLR-Z genus-level taxa) were each summarized by Varimax-rotated PCA and k-means clustering, yielding four dietary patterns and four microbial clusters. A 32-item deficit-accumulation frailty index (FI; frail = FI ≥ 0.21) was the outcome. Regression models adjusted for age, sex and energy intake; a pre-specified mediation model tested whether Microbe PC2 — a butyrate-producer-rich axis dominated by Faecalibacterium, Fusicatenibacter and *Eubacterium eligens* — statistically mediated the fiber–FI association; indirect effects used 5,000-bootstrap percentile 95% CIs.

**Results:**

Frail prevalence was 15.2% overall but varied nine-fold across the 16 diet × microbiota cells, from 4.0% (Pattern 1 [Vegetable, soy and dairy] × Ruminococcus–Faecalibacterium cluster) to 36.4% (Pattern 3 [Seafood and washoku] × Lachnoclostridium–*Ruminococcus gnavus* cluster). Total fiber intake was inversely associated with FI (β = **−**0.142, p < 0.0001), and Microbe PC2 statistically mediated this cross-sectional association (indirect β = **−**0.016, 95% CI **−**0.031 to **−**0.005; Sobel p = 0.009; proportion mediated 11.4%).

**Conclusions:**

Dietary patterns and gut microbial clusters jointly stratify frailty in older Japanese adults, with a small but consistent statistical mediation of the fiber–frailty association via a butyrate-producer-rich microbial axis. Combined dietary–microbial profiling may refine frailty risk assessment.

## Key Points

• Frail prevalence ranged from 4.0% to 36.4% across 16 dietary × microbial cells in 785 older Japanese adults.
• A vegetable–soy–dairy diet combined with a *Ruminococcus*–*Faecalibacterium*-rich gut profile marked the lowest frailty risk.
• Total fiber intake was inversely associated with the frailty index, with ∼11% of the cross-sectional association statistically mediated by a butyrate-producer-rich microbial axis (Microbe PC2).
• Joint dietary–microbial profiling may improve frailty risk stratification beyond either domain alone.

## 1 Introduction

Frailty is a state of decreased physiological reserve and increased vulnerability to stressors that predicts falls, hospitalization, disability and mortality in older adults [1,2]. The deficit-accumulation frailty index (FI) of Rockwood, Mitnitski and Searle expresses frailty as the proportion of age-related deficits accumulated by an individual and provides a continuous risk gradient that complements categorical phenotyping [3]. Identifying modifiable upstream factors — habitual diet and the gut microbiota in particular — is a priority for preventive geriatrics.

Habitual diet is a consistently identified modifiable correlate of frailty. Mediterranean-style patterns and higher intakes of vegetables, fruit, fish, dairy and protein have been linked to lower frailty risk [[4], [5], [6]], and fiber-rich diets to lower inflammation and better physical function [7,8]. Pattern-based approaches such as principal component analysis (PCA) preserve real-world food combinations and outperform nutrient-by-nutrient analysis [9]. In older Japanese, balanced-meal frequency, PCA-derived patterns and dietary variety have each been linked to frailty [[10], [11], [12]], but gut microbiota were not examined. The gut microbiota shifts with age [13], and depletion of butyrate-producing taxa is a hallmark of frailty [14,15]; the NU-AGE Mediterranean intervention shifted the older gut microbiome toward a frailty-protective profile [16]. Whether this putative fiber → microbiota → frailty pathway operates in older Japanese remains unclear.

Kyotango is a Japanese longevity area with one of the highest centenarian densities in the country. A previous study reported that gut microbiota of Kyotango residents were enriched in butyrate-producing taxa compared with an urban Kyoto cohort [17], and a nutrient-level analysis of the Kyotango Multipurpose Cohort found lower plant-protein, micronutrient and fiber intakes and concurrent microbial shifts in frail older adults [18]. Using data from 785 community-dwelling Kyotango residents aged ≥65 years, the present study extends this work with an integrated, pattern-level dietary-microbial framework and a pre-specified mediation model, addressing three objectives: (i) derive interpretable dietary patterns and gut microbial clusters from a 27-item food-group questionnaire and 16S rRNA gene amplicon sequencing; (ii) determine whether the joint 4 × 4 = 16-cell dietary × microbial stratification differentiates frailty more clearly than either domain alone; and (iii) test whether the cross-sectional dietary fiber–frailty association is statistically mediated by a butyrate-producer-rich microbial component (Microbe PC2).

## 2 Materials and methods

### 2.1 Study design and participants

We conducted a cross-sectional analysis using data from the Kyotango Multipurpose Cohort Study, organized by the Department of Longevity and Community Epidemiology, Kyoto Prefectural University of Medicine, which has recruited community-dwelling adults aged ≥65 years from the Kyotango region in northern Kyoto Prefecture. The Kyotango region comprises two cities and two towns with a combined population of approximately 100,000, of whom more than 38% are aged ≥65 years, and contains one of the highest centenarian densities in Japan (>200 centenarians per 100,000 residents) [17]. Of the 798 participants with paired dietary and fecal microbiota data described in our earlier report [18], 6 were excluded for missing frailty-index items and 6 for energy intake outside the range 600–4,000 kcal/day, leaving 786 participants. For the present analysis, one further participant with extensive missing brief-type self-administered diet history questionnaire (BDHQ) data was excluded, yielding a final analytical sample of 785 (Supplementary Figure S1). The study was conducted in accordance with the Declaration of Helsinki and approved by the Ethics and Conflict of Interest Committee of Kyoto Prefectural University of Medicine (approval no. ERB-C-885-4). All participants provided written informed consent.

### 2.2 Dietary assessment and food group construction

Habitual dietary intake during the preceding month was self-reported using the validated brief-type self-administered diet history questionnaire (BDHQ) [19]. The BDHQ estimates intake of foods and nutrients from frequencies of 58 food and beverage items with portion sizes based on standard Japanese references, and its relative validity against 16-day weighed food records has been demonstrated for adults in Japan [19]. To capture habitual dietary patterns at a level coarse enough for stable factor extraction yet fine enough to retain culturally specific food combinations, BDHQ output was aggregated into 27 mutually exclusive food groups by summing intake across conceptually related items (for example, "Soy" combined tofu, fried tofu and natto; "Other vegetables" combined raw lettuce, cabbage and radish/turnip; the full item-to-group mapping is provided in Supplementary Table S1). To remove between-participant variation arising solely from differences in total energy intake, each food group was expressed per 1,000 kcal of total energy intake (density method) [20] and then standardized across participants to mean 0 and standard deviation 1 (Z-score). Total, soluble and insoluble dietary fiber intakes — used as the exposure in the mediation analysis — were energy-adjusted by the same per-1,000-kcal density method, so that all dietary variables in the present study were on a single, internally consistent energy-adjustment framework. This differs from our previous report from the same cohort [18], in which nutrient-level group comparisons used the residual method of Willett [20].

### 2.3 Stool collection, 16S rRNA gene sequencing and taxonomic profiling

Stool samples were self-collected by participants at home, transported under refrigerated conditions and stored at −80 °C until analysis, following the protocol of our previous Kyotango studies [17,18]. Fecal microbial DNA was extracted using the Maxwell® RSC Fecal Microbiome DNA Kit (Promega, Tokyo, Japan) according to the manufacturer's instructions. The V3–V4 region of the 16S rRNA gene was amplified using the primer set 341 F (5′-CCTACGGGNGGCWGCAG-3′) and 805R (5′-GACTACHVGGGTATCTAATCC-3′) with Tks Gflex DNA Polymerase (TaKaRa Bio, Kusatsu, Japan). PCR amplification was performed with the following thermal cycling conditions: an initial denaturation at 95 °C for 3 min, followed by 25 cycles consisting of denaturation at 95 °C for 30 s, annealing at 55 °C for 30 s and extension at 72 °C for 30 s, concluding with a final elongation at 72 °C for 5 min. The resulting amplicons were purified using NucleoFast96 PCR plates (TaKaRa Bio), and a subsequent indexing PCR was carried out using unique dual-index primer sets compatible with MiSeq sequencing, following Illumina’s standard protocol (Illumina, San Diego, CA, USA). After indexing, PCR products were purified, normalized using the SequalPrep Normalization Plate Kit (Life Technologies, Tokyo, Japan), and pooled at equimolar concentrations. The pooled library was purified using AMPure XP magnetic beads (Beckman Coulter, Brea, CA, USA). The resulting purified library was subjected to 285-bp paired-end sequencing on the Illumina MiSeq platform using the MiSeq Reagent Kit v3. Sequencing data were processed as described by Miura et al. [21], with the following exceptions: QIIME 2 [22] version 2024.5 was used for primer trimming, quality filtering, paired-end merging and amplicon sequence variant (ASV) inference with DADA2 [23], and taxonomy of ASVs was assigned against the SILVA 138 reference [24]. ASV counts were collapsed to the genus level, and 47 genus- or family-level taxa with a relative abundance >0.01% in at least 10% of samples were retained for downstream analysis. Because read-derived abundances are compositional, retained taxa were transformed using the centered log-ratio (CLR) transformation after replacement of zero values with a fixed pseudocount of 1.0 × 10^−6^, following the principles of compositional data analysis [25]. CLR values were then standardized to mean 0 and standard deviation 1 across participants (CLR-Z) so that the dietary and microbial feature matrices entered the subsequent principal component analyses on a common scale. Alpha-diversity metrics (Shannon entropy, Chao1 richness and observed features) were computed directly from ASV-level data within QIIME 2 prior to genus collapse and CLR-Z transformation. The raw 16S rRNA gene sequencing data generated in this study have been deposited in the NCBI Sequence Read Archive (SRA) under accession numbers PRJNA1470357 and PRJNA1440982. All other relevant data supporting the findings of this study are included within the article and its supplementary information files (Supplementary Table S2).

### 2.4 Frailty index

Frailty was operationalized using the deficit-accumulation framework of Searle and colleagues [3]. From a 40-item candidate deficit list — covering self-reported symptoms, signs, anthropometric measures, laboratory abnormalities, comorbidities and functional indicators — we retained the 32 items that could be unambiguously coded from the Kyotango Cohort data set, comprising clinical and biochemical measurements, anthropometry, lifestyle and medical-history questionnaire items, and self-rated functional status [18]. The BDHQ (see Dietary assessment and food group construction) was used exclusively for dietary characterization and did not contribute any items to the FI. Each deficit was scored as 0 (absent) or 1 (present), with ordinal items mapped to intermediate values between 0 and 1 (for example, partial impairment scored as 0.5), and the frailty index (FI) was defined as the unweighted sum of deficit scores divided by 32. Following our previous report [18] and established convention [3], FI was analyzed both as a continuous outcome and as a binary categorisation with frailty defined as FI ≥ 0.21. The full deficit list and item-level scoring rules are provided in Supplementary Table S3.

### 2.5 Derivation of dietary patterns and gut microbial clusters

To derive interpretable, pattern-level summaries of habitual diet and gut microbiota, we applied a parallel two-stage procedure to each domain: Varimax-rotated principal component analysis (PCA) followed by k-means clustering of the resulting factor scores.

### 2.6 Factorability and number of components

For each domain, factorability of the standardized feature matrix (785 × 27 for diet; 785 × 47 for microbiota) was assessed by Bartlett's test of sphericity and by the Kaiser–Meyer–Olkin (KMO) measure of sampling adequacy. The number of components retained per domain was determined by jointly considering: (i) the Kaiser criterion of eigenvalue >1 on the unrotated solution; (ii) the inflection point of the scree plot; and (iii) the substantive interpretability of the rotated solution. On this basis, four components were retained for each domain, accounting for cumulative variance explained sufficient for stable downstream clustering (scree plots and variance tables are presented in Supplementary Figure S2 and Supplementary Table S4).

#### 2.6.1 Varimax-rotated PCA

Principal axis factoring was performed using the *factor_analyzer* Python package (method = 'principal'), with orthogonal Varimax rotation [26] applied to the four-component solution, yielding four uncorrelated, simple-structure factors per domain: dietary factors (Food_PC1–PC4) and microbial factors (Microbe_PC1–PC4). Factor loadings with absolute value ≥0.30 were considered salient for interpretation. Factor scores were retained as regression-based standardized scores and exported for clustering and regression analyses.

#### 2.6.2 K-means clustering and cluster labeling

**Fig. 1:**
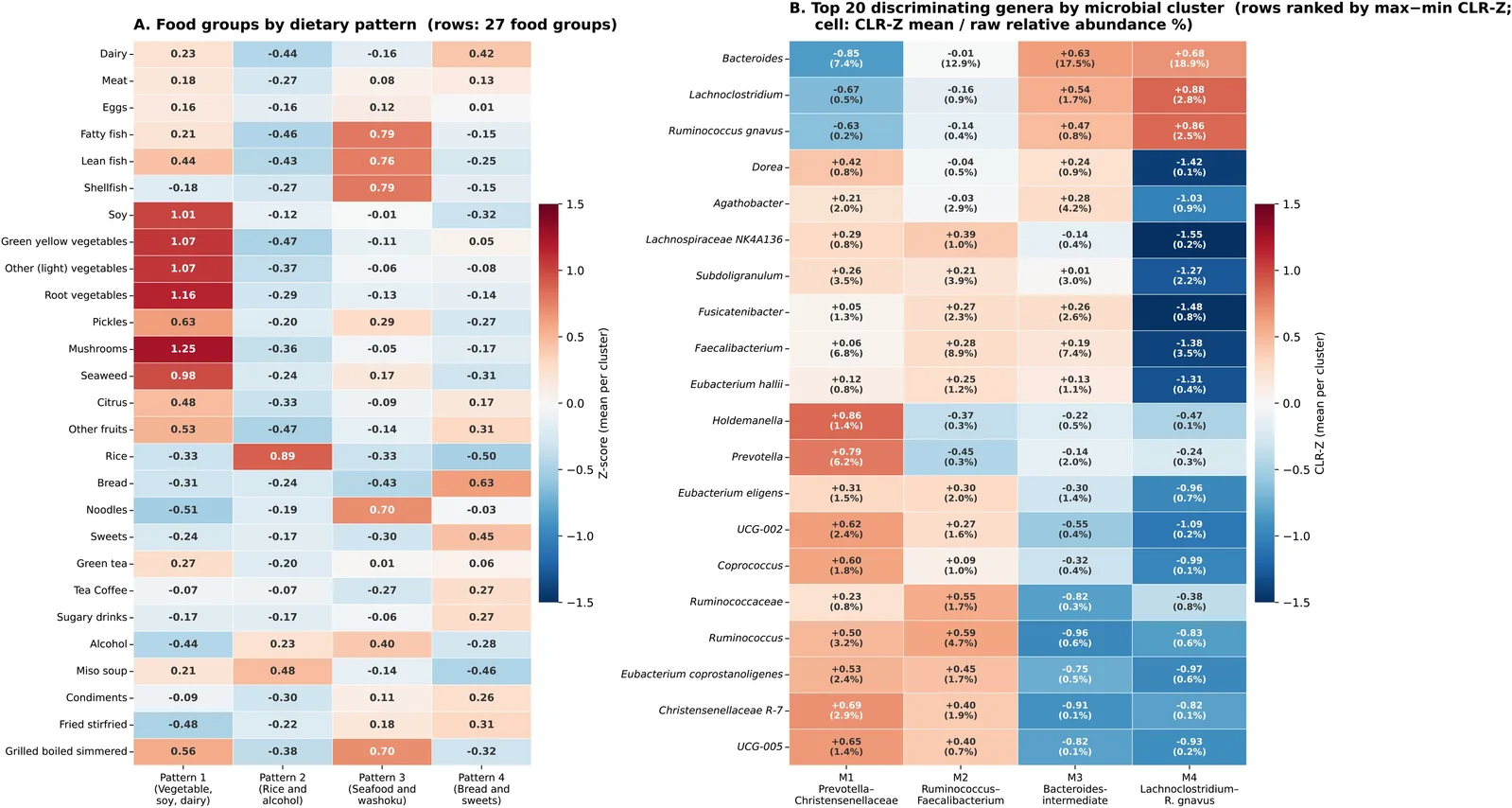
Cluster characterization in 785 community-dwelling Kyotango participants aged ≥65 years. (A) Heatmap of Z-scored mean intake of the 27 BDHQ-derived food groups across the four dietary patterns (Pattern 1, Vegetable, soy and dairy; Pattern 2, Rice and alcohol; Pattern 3, Seafood and *washoku*; Pattern 4, Bread and sweets). (B) Heatmap of mean CLR-Z values for the 20 most discriminating genus- or family-level taxa (ranked by max–min CLR-Z across clusters) across the four gut microbial clusters (*Prevotella*–Christensenellaceae; *Ruminococcus*–*Faecalibacterium*; *Bacteroides*-intermediate; *Lachnoclostridium*–*R. gnavus*). Cell values in panel B show CLR-Z mean / raw relative abundance (%).

For each domain, k-means clustering [27] was applied to the four factor scores with k = 4, random_state = 42 and n_init = 50. The choice of k = 4 was pre-specified for parsimony and consistency between domains, and was confirmed post hoc by inspection of the within-cluster sum of squares (elbow plot) and the average silhouette width across k = 2–8 (Supplementary Figure S3). Cluster centroids in factor-score space and the underlying food-group or genus-level abundance profiles were inspected to assign descriptive labels. Dietary clusters were labelled as Pattern 1 ("Vegetable, soy and dairy"), Pattern 2 ("Rice and alcohol"), Pattern 3 ("Seafood and *washoku*") and Pattern 4 ("Bread and sweets") on the basis of the food groups most strongly elevated at each centroid; microbial clusters were labelled as "*Prevotella*–Christensenellaceae", "*Ruminococcus*–*Faecalibacterium*", "*Bacteroides*-intermediate" and "*Lachnoclostridium*–*R. gnavus*" by the same procedure applied to genus-level CLR-Z values of cluster-defining taxa (Fig. 1 and Supplementary Figure S4). Cluster names are descriptive mnemonics, not exhaustive characterizations.

#### 2.6.3 Composite SCFA-producer score

As an alternative, theory-driven mediator construct used in sensitivity analyses, we computed an unweighted mean of CLR-Z values of six well-established short-chain fatty acid (SCFA)-producing genera — *Faecalibacterium*, *Roseburia*, *Agathobacter*, *Eubacterium hallii* group, *Coprococcus* and *Anaerostipes* — yielding a single composite score ("SCFA_composite") used as a secondary mediator in pre-specified sensitivity analyses.

### 2.7 Statistical analyses and pre-specified mediation model

Baseline characteristics across dietary or microbial clusters were compared by Kruskal–Wallis tests for continuous variables and χ² tests for categorical variables. Independent associations of dietary or microbial cluster membership with frailty were estimated by linear regression of the continuous FI and by logistic regression of the binary frail/non-frail outcome, each adjusted for age, sex and total energy intake. Cluster effects were tested by Type II ANOVA; Pattern 2 ("Rice and alcohol") and the *Ruminococcus*–*Faecalibacterium* cluster were used as numerically largest reference categories. Joint stratification by dietary pattern × microbial cluster was visualised as a 4 × 4 = 16-cell heatmap of cell-specific sample distribution, frail prevalence and mean FI.

The pre-specified mediation hypothesis tested whether Microbe PC2 — the second microbial principal component, characterized by high positive loadings of butyrate-producing taxa including *Faecalibacterium*, *Fusicatenibacter* and the *Eubacterium eligens* group — mediated the association between total dietary fiber intake (exposure, X) and the continuous frailty index (outcome, Y). Following the framework of Baron and Kenny [28] and using the difference method (c − c′), four linear regressions, each adjusted for age, sex and energy intake, were estimated for every exposure–mediator–outcome triplet: the a-path (X → M), b-path (M → Y | X), total effect c (X → Y) and direct effect c′ (X → Y | M). The indirect effect was computed as ab and tested using Sobel's z [29], with 95% percentile confidence intervals for ab obtained from 5,000 nonparametric bootstrap resamples [30]. The proportion mediated was defined as ab/c × 100%. Pre-specified sensitivity analyses substituted soluble fiber or insoluble fiber for total fiber as the exposure, SCFA_composite for Microbe PC2 as the mediator, and binary frail status (logistic outcome) for continuous FI; additional sensitivity analyses were stratified by sex, by age (<75 vs ≥75 years) and excluded the smallest dietary cluster to assess robustness.

All analyses were performed in Python 3.11 using pandas, NumPy, scikit-learn (PCA and KMeans), *factor_analyzer* (Varimax rotation), statsmodels (linear and logistic regression, Type II ANOVA) and SciPy (distributional tests). Baseline descriptive comparisons were repeated using JMP Pro 16 (SAS Institute Japan), consistent with our previous report [18]. Two-sided *p* < 0.05 was considered statistically significant. As primary 4-cluster and 16-cell analyses were pre-specified, no formal multiple-comparison correction was applied; this limitation is acknowledged in the Discussion.

## 3 Results

### 3.1 Participant characteristics

Of 798 community-dwelling Kyotango residents aged ≥65 years with paired BDHQ and fecal microbiota data, 13 were excluded according to the criteria detailed in Methods, leaving 785 participants in the analytic sample. Median age was 72 years (interquartile range, IQR 69–76); 469 (59.7%) were women. Mean (SD) energy intake was 1,878 (550) kcal/day. The frailty index (FI) ranged from 0 to 0.50, with a median of 0.121 (IQR 0.083–0.172) and a mean (SD) of 0.137 (0.075); 119 participants (15.2%) were classified as frail (FI ≥ 0.21). Baseline characteristics are summarized in Table 1.

**Table 1:** Baseline characteristics of 785 community-dwelling Kyotango participants aged ≥65 years, by frailty status.

**Variable**	**Non-frail (n = 666)**	**Frail (n = 119)**	**p value**	**Test**
Age (years)	71.0 (69.0–75.0)	76.0 (70.0–81.0)	**<0.0001**	MWU
Sex (Female)	399 (59.9%)	70 (58.8%)	0.900	χ^2^
Height (cm)	156.5 (151.0–162.5)	153.4 (146.8–160.9)	**0.002**	MWU
Weight (kg)	56.0 (50.1–62.9)	58.6 (51.0–65.0)	0.140	MWU
MMSE total	28.0 (26.0–29.0)	26.0 (24.0–28.0)	**<0.0001**	MWU
MCI (yes)	203 (44.0%)	51 (69.9%)	**<0.0001**	χ^2^
Sarcopenia (yes)	40 (6.0%)	22 (18.5%)	**<0.0001**	χ^2^
BMI ≥25 (yes)	165 (24.8%)	51 (42.9%)	**<0.0001**	χ^2^
Large waist circ. (yes)	243 (36.7%)	62 (52.5%)	**0.002**	χ^2^
Obesity (yes)	165 (24.8%)	51 (42.9%)	**<0.0001**	χ^2^
Brain disease	28 (4.2%)	4 (3.4%)	0.810	Fisher
Heart disease	59 (8.9%)	14 (11.8%)	0.400	χ^2^
GI disease	131 (19.7%)	15 (12.6%)	0.090	χ^2^
Liver disease	42 (6.3%)	5 (4.2%)	0.500	χ^2^
Kidney disease	26 (3.9%)	5 (4.2%)	1.000	χ^2^
Atopic dermatitis	12 (1.8%)	5 (4.2%)	0.190	χ^2^
Bronchial asthma	30 (4.5%)	12 (10.1%)	**0.020**	χ^2^
Diabetes mellitus	70 (10.5%)	14 (11.8%)	0.810	χ^2^
Hypertension	249 (37.4%)	47 (39.5%)	0.740	χ^2^
Lipid disorder	207 (31.1%)	33 (27.7%)	0.530	χ^2^
Urethral stone	44 (6.6%)	3 (2.5%)	0.090	Fisher
Dementia	8 (1.2%)	1 (0.8%)	1.000	Fisher
Rheumatoid arthritis	7 (1.1%)	3 (2.5%)	0.180	Fisher
History of cancer	56 (8.4%)	19 (16.0%)	**0.020**	χ^2^
Chronic disease (n)	1.0 (0.0–2.0)	1.0 (0.0–2.0)	0.850	MWU
Multimorbidity (≥2)	267 (40.1%)	52 (43.7%)	0.520	χ^2^
Education ≥ high school	480 (72.1%)	94 (79.0%)	0.150	χ^2^
Hearing loss	435 (65.5%)	80 (67.2%)	0.800	χ^2^
Head injury history	3 (0.5%)	0 (0.0%)	1.000	Fisher
Visual impairment	67 (10.2%)	26 (22.0%)	**0.0005**	χ^2^
Current smoking	172 (25.9%)	33 (27.7%)	0.750	χ^2^
Current alcohol	264 (39.8%)	43 (36.4%)	0.560	χ^2^
Polypharmacy (≥5)	24 (3.6%)	24 (20.2%)	**<0.0001**	χ^2^
Constipation	223 (33.5%)	48 (40.3%)	0.180	χ^2^
Has housemate	587 (88.1%)	101 (84.9%)	0.400	χ^2^
Has spouse	518 (77.9%)	86 (72.3%)	0.220	χ^2^
Regular exercise	279 (41.9%)	63 (52.9%)	**0.030**	χ^2^
Depression	105 (15.8%)	67 (56.3%)	**<0.0001**	χ^2^
Total protein (g/dL)	7.20 (6.90–7.40)	7.30 (6.90–7.50)	0.060	MWU
Albumin (g/dL)	4.20 (4.00–4.40)	4.20 (4.00–4.40)	0.600	MWU
Fisher ratio	3.20 (2.87–3.55)	3.28 (2.93–3.55)	0.610	MWU
AST (U/L)	22.0 (19.0–26.0)	22.0 (18.0–27.0)	0.880	MWU
ALT (U/L)	18.0 (15.0–23.0)	18.0 (14.0–25.0)	0.790	MWU
Total cholesterol (mg/dL)	210.0 (188.0–233.8)	194.0 (172.5–218.0)	**<0.0001**	MWU
LDL-C (mg/dL)	124.0 (106.0–147.0)	109.0 (89.5–132.0)	**<0.0001**	MWU
HDL-C (mg/dL)	67.0 (55.0–80.0)	63.0 (53.0–73.5)	**0.020**	MWU
Triglyceride (mg/dL)	103.0 (76.0–143.8)	114.0 (80.0–163.5)	0.130	MWU
eGFR	69.7 (60.6–77.4)	65.9 (54.0–74.9)	**0.004**	MWU
Cystatin-C eGFR	90.8 (82.5–100.5)	83.1 (66.5–93.2)	**<0.0001**	MWU
Fasting glucose (mg/dL)	100.0 (95.0–106.0)	101.0 (96.5–111.0)	0.090	MWU
HbA1c (%)	5.70 (5.50–5.90)	5.80 (5.50–6.15)	**0.030**	MWU
WBC (×10²/μL)	57.8 (49.6–67.6)	58.8 (50.0–71.1)	0.250	MWU
Lymphocyte (%)	34.1 ± 7.9	33.0 ± 8.2	0.170	t
Neutrophil/Lymphocyte	0.59 (0.46–0.74)	0.55 (0.41–0.70)	0.130	MWU
RBC (×10⁴/μL)	432.0 (406.0–459.0)	433.0 (399.0–455.5)	0.530	MWU
MCV (fL)	93.1 (90.5–95.9)	92.7 (90.0–95.8)	0.280	MWU
RDW (%)	12.7 (12.2–13.2)	12.9 (12.3–13.2)	**0.030**	MWU
Ferritin (ng/mL)	102.0 (63.1–161.0)	84.1 (50.2–142.5)	0.050	MWU
25(OH) Vitamin D (ng/mL)	21.8 (17.7–27.0)	23.2 (16.1–28.9)	0.260	MWU
IL-6 (pg/mL)	1.24 (0.90–1.87)	1.45 (1.13–2.06)	**0.0007**	MWU
HS-CRP (mg/dL)	0.041 (0.022–0.077)	0.050 (0.027–0.114)	**0.020**	MWU

Values are median (interquartile range) for continuous variables and n (%) for categorical variables. Frailty was defined as a frailty index ≥0.21 (deficit accumulation method; 32 of 40 candidate items, Searle et al.). MWU, Mann–Whitney U test; χ^2^, chi-squared test.

### 3.2 Derivation of dietary patterns and gut microbial clusters

#### 3.2.1 Dietary patterns

Varimax-rotated PCA on the 27 energy-adjusted food groups extracted four interpretable factors that together explained 36.2% of variance in the unrotated solution and showed simple structure after rotation (Supplementary Figure S2A, Supplementary Table S4). Food_PC1 was characterized by high loadings of root and other vegetables, mushrooms, soy, green and yellow vegetables, and seaweed; Food_PC2 by Japanese condiments, fried/stir-fried dishes and meat with a negative loading on rice; Food_PC3 by dairy, other fruits, bread and sweets with negative loadings on rice and miso soup; and Food_PC4 by lean and fatty fish, shellfish and grilled/boiled/simmered preparations. K-means clustering on these factor scores identified four dietary patterns (Pattern 1, *n* = 114, 14.5%; Pattern 2, *n* = 248, 31.6%; Pattern 3, *n* = 162, 20.6%; Pattern 4, *n* = 261, 33.2%). Pattern centroids supported the descriptive labels (Fig. 1A and Supplementary Figure S4A): Pattern 1 — high Food_PC1 (Vegetable, soy and dairy); Pattern 2 — low across all factors, with the most negative Food_PC3 (Rice and alcohol); Pattern 3 — high Food_PC4 (Seafood and *washoku*); and Pattern 4 — high Food_PC3 (Bread and sweets).

#### 3.2.2 Gut microbial clusters

Varimax-rotated PCA on the 47 CLR-Z genus- and family-level taxa extracted four interpretable factors explaining 35.3% of unrotated variance (Supplementary Figure S2B, Supplementary Table S4; the full 47-taxa CLR-Z heatmap stratified by microbial cluster is provided in Supplementary Figure S6). Microbe_PC1 contrasted *Bacteroides*, *Blautia*, *Lachnoclostridium*, Lachnospiraceae and the *Ruminococcus* gnavus group (positive) against *Christensenellaceae* R-7 group, UCG-002/UCG-005, *Ruminococcus* and *Prevotella* (negative); Microbe_PC2 was dominated by positive loadings of butyrate-producing taxa — *Fusicatenibacter*, *Faecalibacterium*, Lachnospiraceae NK4A136 group, *Eubacterium hallii* group, *Subdoligranulum*, *Agathobacter*, *Roseburia* and *Eubacterium eligens* group, with negative loadings of *Lactobacillus* and *Klebsiella*; Microbe_PC3 reflected Ruminococcaceae, *Christensenellaceae* R-7 group, *Alistipes* and *Akkermansia*; and Microbe_PC4 captured *Phascolarctobacterium* with a negative loading on *Dialister*. K-means clustering yielded four microbial clusters (*Prevotella*–Christensenellaceae, *n* = 218, 27.8%; *Ruminococcus*–*Faecalibacterium*, *n* = 274, 34.9%; *Bacteroides*-intermediate, *n* = 201, 25.6%; *Lachnoclostridium*–*R. gnavus*, *n* = 92, 11.7%); centroid profiles are shown in Fig. 1B and Supplementary Figure S4B. The *Ruminococcus*–*Faecalibacterium* cluster was characterized by the highest Microbe_PC2 score, and the *Lachnoclostridium*–*R. gnavus* cluster by the highest Microbe_PC1 score and the lowest Microbe_PC2 score.

### 3.3 Independent associations of dietary patterns and microbial clusters with frailty

**Fig. 2:**
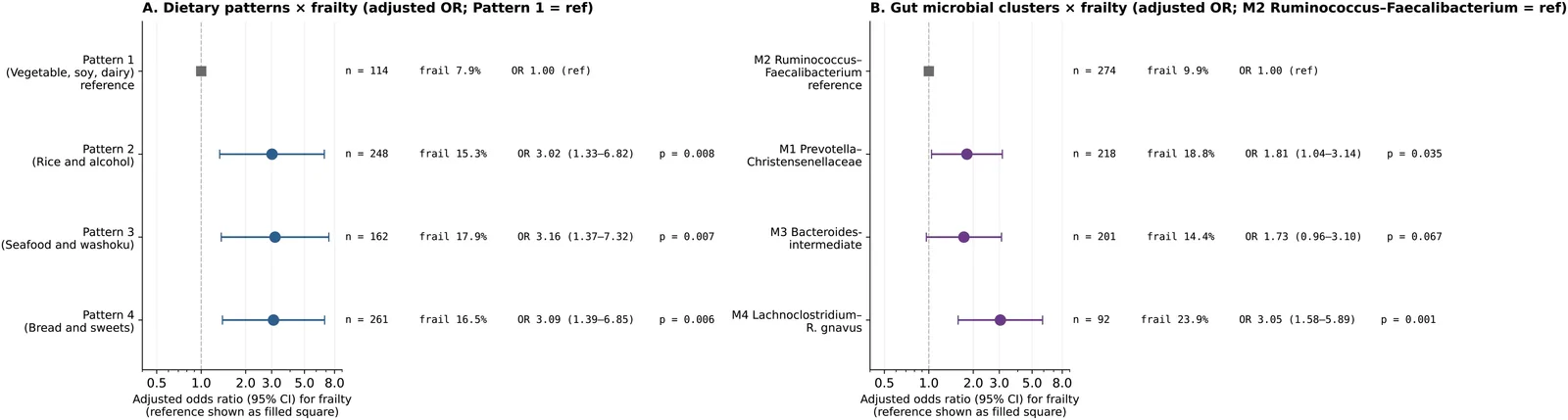
Forest plot of adjusted odds ratios for frailty (FI ≥ 0.21) across (A) dietary patterns and (B) microbial clusters. Models adjusted for age, sex, BMI and total energy intake. Reference categories: Pattern 1 (Vegetable, soy and dairy) for dietary patterns; *Ruminococcus*–*Faecalibacterium* cluster for microbial clusters.

Crude frail prevalence varied across dietary patterns from 7.9% (9/114) in Pattern 1 (Vegetable, soy and dairy) to 17.9% (29/162) in Pattern 3 (Seafood and *washoku*), and across microbial clusters from 9.9% (27/274) in the *Ruminococcus*–*Faecalibacterium* cluster to 23.9% (22/92) in the *Lachnoclostridium*–*R. gnavus* cluster (Table 1; Fig. 2). Adjusted analyses (Fig. 2; Supplementary Table S5) confirmed these gradients. With Pattern 1 as the reference and adjustment for age and sex, the adjusted odds ratios (aORs) of frailty were 3.13 (95% CI 1.39–7.06; *p* = 0.006) for Pattern 2, 3.39 (1.48–7.78; *p* = 0.004) for Pattern 3 and 3.28 (1.49–7.24; *p* = 0.003) for Pattern 4; on the continuous FI, the Type II ANOVA *p* for dietary cluster was 0.003 (F₃3,779 = 4.82). With the *Ruminococcus*–*Faecalibacterium* cluster as the reference, the aOR was 1.90 (1.11–3.27; *p* = 0.020) for the *Prevotella*–Christensenellaceae cluster, 1.77 (0.99–3.15; *p* = 0.054) for the *Bacteroides*-intermediate cluster and 3.00 (1.57–5.73; *p* = 0.001) for the *Lachnoclostridium*–*R. gnavus* cluster (omnibus *p* = 0.009; F₃3,779 = 3.88). Further adjustment for BMI and energy intake left effect sizes essentially unchanged (Pattern 2 aOR 3.02 [1.34–6.82], Pattern 4 aOR 3.09 [1.39–6.85]; *Lachnoclostridium*–*R. gnavus* aOR 3.05 [1.58–5.89]; Supplementary Table S5).

### 3.4 Joint stratification of frailty by diet × microbiota: 16-cell analysis

**Fig. 3:**
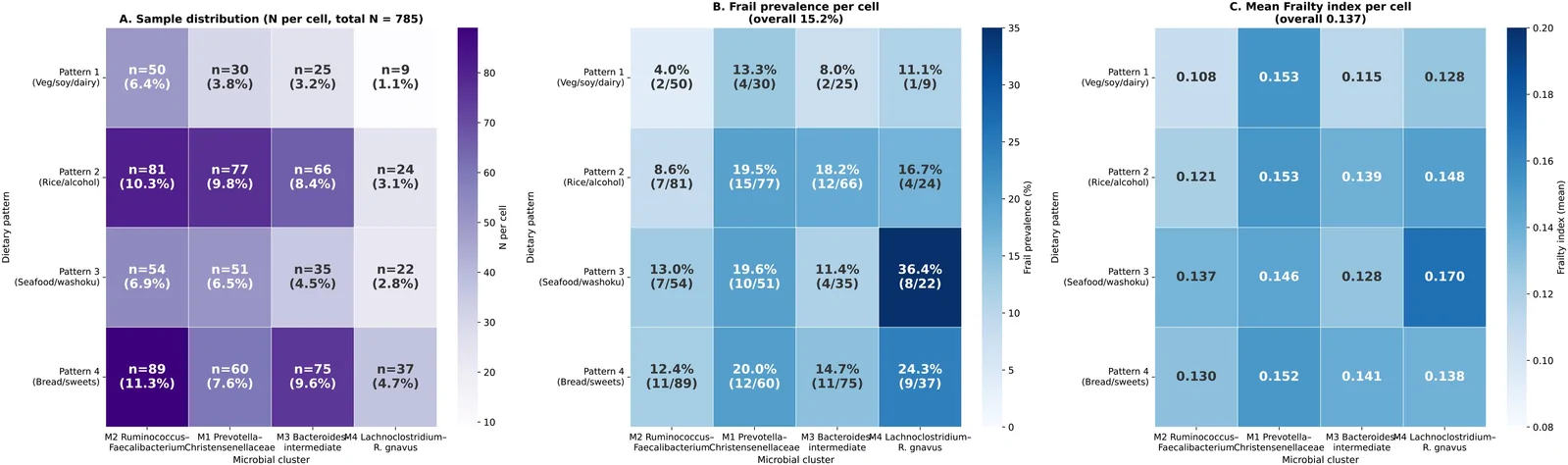
Joint 4 × 4 = 16-cell stratification of frailty by dietary pattern × microbial cluster. (A) Sample distribution (N per cell); (B) frail prevalence (FI ≥ 0.21); (C) mean frailty index per cell.

Cross-classifying the 4 × 4 = 16 dietary × microbial cells revealed substantially greater heterogeneity in frailty than either domain alone (Fig. 3). Cell-level sample sizes ranged from *n* = 9 to *n* = 89, with the four largest cells (Pattern 4 × *Ruminococcus*–*Faecalibacterium*; Pattern 2 × *Ruminococcus*–*Faecalibacterium*; Pattern 2 × *Prevotella*–Christensenellaceae; Pattern 4 × *Bacteroides*-intermediate) jointly containing 41.7% of the cohort. Across cells, frail prevalence varied more than nine-fold, from 4.0% (2/50) in Pattern 1 (Vegetable, soy and dairy) paired with the *Ruminococcus*–*Faecalibacterium* cluster to 36.4% (8/22) in Pattern 3 (Seafood and *washoku*) paired with the *Lachnoclostridium*–*R. gnavus* cluster (Fig. 3B). Mean FI showed a parallel gradient, from 0.108 to 0.170 (Fig. 3C). The two cells with the highest frail prevalence (Pattern 3 × *Lachnoclostridium*–*R. gnavus*, 36.4%; Pattern 4 × *Lachnoclostridium*–*R. gnavus*, 24.3%) and the two with the lowest (Pattern 1 × *Ruminococcus*–*Faecalibacterium*, 4.0%; Pattern 1 × *Bacteroides*-intermediate, 8.0%) revealed a consistent pattern in which the *Lachnoclostridium*–*R. gnavus* cluster appeared to amplify frailty risk across dietary patterns, while the *Ruminococcus*–*Faecalibacterium* cluster attenuated it. As the smallest cells contained as few as nine participants, exact cell-level percentages should be interpreted alongside cell *N* (Fig. 3A).

### 3.5 Dietary fiber, Microbe PC2 and the frailty index: pre-specified mediation analysis

**Fig. 4:**
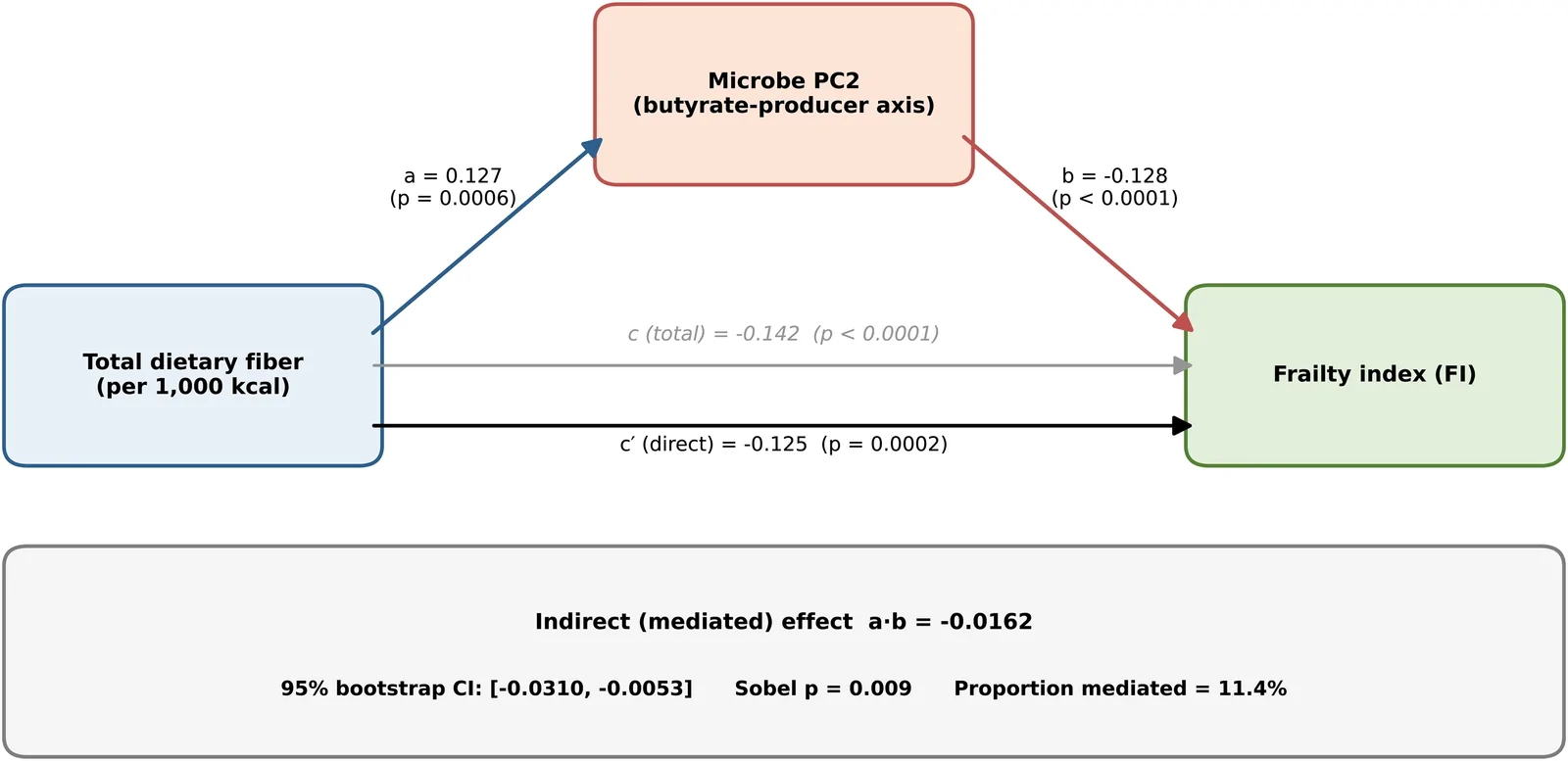
Path diagram of the pre-specified linear mediation model: total dietary fiber intake → Microbe PC2 → frailty index. Standardized path coefficients (a, b, c, c′) and the bootstrap 95% CI for the indirect effect ab are shown; covariates were age, sex and total energy intake.

In linear regressions adjusted for age, sex and energy intake, total dietary fiber intake (per 1,000 kcal) was inversely associated with the continuous FI (standardized β = −0.142, *p* < 0.0001; Table 2). Total dietary fiber was positively associated with Microbe_PC2, the butyrate-producer-rich axis (a-path β = 0.127, *p* = 0.0006), and Microbe_PC2 was, in turn, inversely associated with the FI adjusted for fiber (b-path β = −0.128, *p* < 0.0001). The indirect (mediated) effect was **−**0.016 (95% bootstrap percentile CI **−**0.031 to −0.005; 5,000 resamples), with Sobel *z* corresponding to *p* = 0.009 and an estimated proportion mediated of 11.4%; the direct effect remained significant (β = −0.125, *p* = 0.0002), consistent with partial mediation (Table 2; Fig. 4). Substituting soluble fiber (indirect β = −0.013, 95% CI −0.027 to −0.003; proportion mediated 10.3%) or insoluble fiber (indirect β = −0.016, 95% CI −0.030 to −0.005; proportion mediated 11.1%) for total fiber gave essentially congruent results (Table 2). Using the theory-driven SCFA composite as an alternative mediator produced a smaller indirect effect that did not consistently reach significance (indirect β for total fiber = −0.008, 95% CI −0.018 to −0.001; Sobel *p* = 0.06; proportion mediated 5.9%), suggesting that the data-driven Microbe_PC2 captures more frailty-relevant SCFA-producer signal than the six-genus composite. Pre-specified sensitivity analyses (Supplementary Table S6) showed congruent point estimates in women (indirect β = −0.016, 95% CI −0.036 to **−**0.002; Sobel *p* = 0.05; proportion mediated 15.7%), in participants aged <75 years (indirect β = −0.014; Sobel *p* = 0.05; proportion mediated 14.5%) and after excluding the smallest dietary cluster (Pattern 1; indirect β = −0.014, 95% CI **−**0.033 to 0.000; Sobel *p* = 0.07); in men and in those aged ≥75 years, bootstrap CIs crossed zero, reflecting reduced power in these subgroups. A complementary logistic mediation model with binary frail status as the outcome gave a total-effect log-odds of −0.498 (OR 0.61, *p* < 0.0001) per SD increase in total fiber, with an indirect log-odds effect of −0.042 (95% CI −0.085 to −0.011; Sobel *p* = 0.02), corroborating the continuous-FI findings (Supplementary Table S7).

**Table 2:** Pre-specified linear mediation models: dietary fiber → microbial axis → frailty index in 785 community-dwelling Kyotango participants aged ≥65 years.

**Model**	**Exposure (X)**	**Mediator (M)**	**a (X→M) β [p]**	**b (M→Y) β [p]**	**c (total) β [p]**	**c′ (direct) β [p]**	**Indirect ab**	95% **bootstrap CI**	**Sobel p**	**Proportion mediated (%)**
**M1**	**Total fiber**	**Microbe PC2**	**0.127 [0.0006]**	**−0.128 [<0.0001]**	**−0.142 [<0.0001]**	**−0.125 [0.0002]**	**−0.0162**	**[-0.0310, −0.0053]**	**0.009**	**11.4**
M2	Soluble fiber	Microbe PC2	0.101 [0.007]	−0.132 [<0.0001]	−0.129 [0.0001]	−0.116 [0.0006]	−0.0132	[-0.0274, **−**0.0029]	0.02	10.3
M3	Insoluble fiber	Microbe PC2	0.122 [0.0009]	−0.128 [<0.0001]	−0.141 [<0.0001]	−0.126 [0.0002]	−0.0157	[-0.0304, **−**0.0048]	0.01	11.1
M4	Total fiber	SCFA composite	0.089 [0.02]	−0.094 [0.003]	−0.142 [<0.0001]	−0.133 [<0.0001]	−0.0084	[-0.0184, **−**0.0012]	0.06	5.9
M5	Soluble fiber	SCFA composite	0.069 [0.06]	−0.097 [0.002]	−0.129 [0.0001]	−0.122 [0.0003]	−0.0067	[-0.0162, 0.0001]	0.11	5.2
M6	Insoluble fiber	SCFA composite	0.084 [0.02]	−0.094 [0.003]	−0.141 [<0.0001]	−0.133 [<0.0001]	−0.008	[-0.0180, **−**0.0008]	0.07	5.6

Models M1–M3 use Microbe PC2 (a butyrate-producer-rich axis) as mediator; M4–M6 substitute the six-genus SCFA composite as an alternative mediator. All models adjusted for age, sex and energy intake. β are standardized path coefficients; the indirect effect ab uses the difference method (c–c′) with 95% percentile CI from 5,000 bootstrap resamples; Sobel p is from the standard Sobel z test; proportion mediated = ab/c × 100%.

## 4 Discussion

In 785 community-dwelling Kyotango residents aged ≥65 years, habitual dietary patterns and gut microbial clusters each independently differentiated frailty risk, and their joint 4 × 4 stratification revealed a more than nine-fold gradient in frail prevalence — from 4.0% in a vegetable–soy–dairy diet paired with a *Ruminococcus*–*Faecalibacterium* cluster to 36.4% in a seafood-and-*washoku* diet paired with a *Lachnoclostridium*–*R. gnavus* cluster — that neither domain captured alone. Pattern 1 ("Vegetable, soy and dairy") was the lowest-frailty dietary pattern; the three contrasting patterns each showed an approximately three-fold higher adjusted odds of frailty after adjustment for age, sex, BMI and energy intake. The *Lachnoclostridium*–*R. gnavus* microbial cluster carried the highest microbial risk (adjusted OR 3.0 vs the *Ruminococcus*–*Faecalibacterium* cluster), whereas a *Ruminococcus*–*Faecalibacterium*-rich profile was protective regardless of dietary pattern. A pre-specified mediation analysis indicated that approximately 11% of the inverse association between total dietary fiber intake and the continuous frailty index was statistically mediated by Microbe PC2 (cross-sectional mediation) — a data-driven microbial axis dominated by butyrate-producing taxa including *Faecalibacterium*, *Fusicatenibacter* and the *Eubacterium eligens* group — with congruent direction across soluble and insoluble fiber and across binary frailty outcomes.

Our findings align with, and extend, three streams of prior research. First, in older Japanese, the frequency of balanced *washoku*-style meals [10], a posteriori PCA-derived dietary patterns [11] and dietary variety [12] have each been linked to frailty, but none of these studies examined gut microbiota concurrently. The present study confirms that diet differentiates frailty in older Japanese adults but adds two key observations: (i) the lowest-frailty pattern in our cohort was vegetable-, soy- and dairy-rich rather than the seafood-and-*washoku* pattern that traditional Japanese dietary scores would prioritise; and (ii) within each dietary pattern, microbial composition further re-stratified frailty risk. The protective profile of Pattern 1 echoes the NU-AGE Mediterranean intervention finding that increased plant, dairy and pulse intake shifted the older gut microbiome toward an anti-inflammatory, frailty-protective profile [16].

A second, perhaps unexpected, observation deserves comment: Pattern 3 ("Seafood and *washoku*") — characterized by high intake of lean and fatty fish, shellfish and grilled/boiled/simmered preparations — was not protective relative to Pattern 1, and in fact carried an adjusted odds ratio of frailty comparable to Patterns 2 and 4. Nutrient comparisons across the four dietary patterns provide a plausible explanation (Supplementary Table S8). Pattern 3 had the highest sodium intake (2,868 mg/1,000 kcal) and the highest salt-equivalent intake (7.2 g/1,000 kcal) of any pattern, with a sodium-to-potassium ratio of 1.89 — only marginally below Pattern 2 (1.87) and substantially higher than the favorable Pattern 1 (1.30). High dietary salt has been shown to deplete intestinal *Lactobacillus* and to expand pro-inflammatory T-cell responses in both murine models and a human pilot study [33], providing a plausible diet → microbiota → inflammation pathway by which a seafood-and-*washoku* pattern that is heavy in salted, pickled and simmered preparations may blunt the otherwise protective elements of traditional Japanese foods. Consistent with this interpretation, direct comparison of the four dietary patterns in this cohort (Supplementary Table S8) showed that Pattern 3 carried the highest sodium intake (2,868 mg/1,000 kcal), the highest salt-equivalent intake (7.2 g/1,000 kcal) and a high sodium-to-potassium ratio (1.89), while Pattern 1 carried the highest potassium intake (2,151 mg/1,000 kcal) and the lowest sodium-to-potassium ratio (1.28). Pattern 1 stood out at the opposite pole, with the highest potassium intake (2,151 mg/1,000 kcal), the highest total dietary fiber (10.5 g/1,000 kcal), the highest calcium and magnesium, the highest plant-protein intake and the lowest Na/K ratio — a nutrient signature more reminiscent of a DASH-style or NU-AGE Mediterranean profile [16] than of any single traditional Japanese dietary score. Importantly, although we describe Pattern 1 as “vegetable, soy and dairy”, we do not attribute its protective association solely to dietary-fiber content: ordinary dairy products contain little or no fiber and it would be misleading to interpret this pattern as fiber-rich purely on that basis. Rather, dairy contributes to Pattern 1 through a coherent, multi-nutrient signature — high calcium, magnesium, potassium and animal- and plant-protein density coupled with low Na/K ratio — that runs in parallel to the plant-food (vegetables, soy) component that provides most of the fiber and polyphenols. This multi-nutrient interpretation is consistent with the observation that only ∼11% of the cross-sectional fiber–frailty association was statistically mediated by Microbe PC2, indicating that non-microbial pathways (for example, direct effects of protein, calcium, potassium and magnesium on musculoskeletal and cardiovascular determinants of frailty) contribute the majority of the effect.

Our results also build directly on our previous nutrient-level report from the same cohort [18]. That earlier analysis identified lower intakes of plant protein, several micronutrients and dietary fiber in the frail group and reported correlations between fiber intake and butyrate-producing taxa, but did not formally examine pattern-level diet × microbiota interactions nor a pre-specified mediation model. By moving from nutrients to data-driven dietary patterns, and from bivariate correlations to a 4 × 4 cluster matrix linked through a single-mediator model, the present study provides an integrated, mechanism-orientated re-analysis of the same Kyotango source data.

Finally, in the broader gut–frailty literature, the depletion of butyrate-producing taxa in frail older adults observed in the ELDERMET [14] and NU-AGE [16] cohorts of European long-term-care or community-dwelling adults is recapitulated in our Kyotango sample: the cluster most enriched in Microbe PC2 was associated with the lowest frailty across dietary contexts. Our work therefore suggests that the diet–microbiota–frailty axis described in European populations operates in older Japanese adults despite distinct dietary and microbial baselines, supporting biological generalisability of this axis across two contrasting food cultures.

The biological plausibility of Microbe PC2 as a partial mediator rests on the central role of short-chain fatty acids, and butyrate in particular, in host physiology relevant to aging. Butyrate is the preferred energy substrate of colonocytes, reinforces gut-barrier integrity and modulates systemic inflammation through GPR41/43-mediated signalling and histone-deacetylase inhibition [31]. Conceptual frameworks for a "gut–muscle axis" propose that age-related decline in butyrate-producing bacteria contributes to sarcopenia and frailty through chronic low-grade inflammation, anabolic resistance and impaired energy-substrate availability [32]. Within Microbe PC2, the dominant positive loadings — *Faecalibacterium*, *Fusicatenibacter*, *Eubacterium eligens* group, *Roseburia*, *Agathobacter* and *Subdoligranulum* — are all well-characterized SCFA producers, and the negative loadings on *Lactobacillus* and Enterobacteriaceae are consistent with a healthy, low-pathobiont profile. Our finding that the data-driven Microbe PC2 yielded a stronger and more robust indirect effect than a six-genus theory-driven SCFA composite is consistent with PC2 capturing a broader, coherent ecological signal — including covariance among SCFA producers and inversely loading pathobionts — than any pre-defined panel.

A complementary interpretation arises from the contrasting microbial cluster at the opposite end of Microbe PC2: the *Lachnoclostridium*–*R. gnavus* cluster was enriched in taxa associated with chronic inflammation, mucin degradation and metabolic disturbance, most notably *Ruminococcus gnavus* and *Lachnoclostridium*. The persistent ∼3-fold higher adjusted odds of frailty in this cluster across dietary patterns suggests that an unfavorable microbiota may attenuate the dietary benefits attainable through food choice alone, although causal inference requires prospective and interventional data.

A further biologically relevant signal outside the primary mediation axis deserves mention. Microbe PC3, although not selected as the primary mediator, was dominated by positive loadings of *Akkermansia* alongside Ruminococcaceae, *Christensenellaceae* R-7 group and *Alistipes*. This axis warrants dedicated comment because *Akkermansia muciniphila* has emerged as a key microbial marker of healthy aging, with several reports of enrichment in centenarians and long-lived populations and of favorable associations with metabolic homeostasis, gut-barrier integrity and immune regulation. Kyotango is a nationally recognized longevity area with a distinctive gut microbial ecosystem, so the prominence of *Akkermansia* in Microbe PC3 may reflect an additional microbial signature of healthy aging that is complementary to, and mechanistically distinct from, the butyrate-producer-rich axis captured by Microbe PC2. Although the mediation effect through Microbe PC3 did not reach statistical significance in the present pre-specified analysis, its biological relevance warrants further investigation. *Akkermansia* has also been implicated in disorders of gut-brain interaction (DGBI), including functional dyspepsia and irritable bowel syndrome, through effects on mucosal barrier function, immune regulation and microbial metabolite production that shape visceral sensitivity and gastrointestinal symptom generation. Given the recognized overlap between frailty, malnutrition, gastrointestinal symptoms and DGBI in older adults, it is plausible that *Akkermansia*-related pathways participate in a broader gut–brain–muscle axis linking aging, digestive function and physical resilience. Longitudinal studies incorporating symptom-based DGBI assessments together with shotgun metagenomics and metabolomic profiling will be required to determine whether *Akkermansia* represents a marker or a mediator of healthy aging and resistance to frailty.

Strengths of this study include a relatively large, community-dwelling Japanese cohort with paired BDHQ-derived dietary data and 16S rRNA gene-based microbiota profiling; a deficit-accumulation frailty index based on 32 of 40 candidate items, scored consistently across participants and identically to our previous report from this cohort [18]; orthogonal Varimax-rotated PCA combined with k-means clustering at parallel resolution in both dietary and microbial domains; CLR transformation appropriate for compositional microbiome data; and a pre-specified mediation analysis with bootstrap percentile confidence intervals across multiple exposure and mediator definitions.

Several limitations should temper interpretation. First, and most importantly, the cross-sectional design precludes causal inference. Our mediation analysis should therefore be interpreted as demonstrating statistical (cross-sectional) mediation rather than evidence of a causal biological pathway, because the temporal ordering required for causal mediation cannot be established when exposure, mediator and outcome are measured simultaneously. Reverse causation therefore remains an equally plausible explanation: frail individuals may modify their dietary habits (for example, reducing fiber-rich foods because of chewing difficulty, altered appetite, or dietary restriction related to comorbidities), and frailty-related physiological changes, chronic inflammation, altered gut motility, or reduced food diversity may themselves alter gut microbial composition, thereby producing the same statistical mediation pattern in the opposite direction. Longitudinal cohorts capturing time-ordered exposure, mediator and outcome measurements and, ultimately, dietary interventional trials will be required to establish temporal sequence and to test whether the fiber → microbiota → frailty pathway is causal. Second, dietary intake was self-reported via the BDHQ; although the BDHQ has been validated against weighed food records in Japanese adults [19], food-frequency instruments carry residual measurement error that may attenuate associations. Third, 16S rRNA gene amplicon sequencing limits taxonomic resolution to the genus level and yields only indirect inference about functional (e.g. SCFA-producing) capacity; fecal SCFA quantification, shotgun metagenomics, and metabolomic profiling would provide more direct mechanistic evidence in future studies. Fourth, although the overall sample of 785 was substantial, some of the 16 joint cells contained as few as nine participants, so individual cell-level frail percentages should be read alongside cell *N* (Fig. 3A). Fifth, the mediation analysis in male strata was underpowered; the apparent stronger mediation in women may reflect a true sex-specific effect, larger female sample size or unmeasured confounders, and warrants confirmation. Sixth, we did not formally adjust for multiple comparisons in the primary 4-cluster and 16-cell analyses; this was a deliberate pre-specified choice consistent with the hypothesis-driven structure but invites caution in interpreting cell-level extremes. Seventh, individual medication records beyond a polypharmacy flag (≥5 concurrent medications) were captured only for proton pump inhibitors (PPIs, n = 40, 5.1%) and systemic antibiotics (n = 5, 0.6%), and full prescription-level information (for example, use of metformin, statins, laxatives or opioids) was not available. Sensitivity analyses excluding PPI or antibiotic users, or including these variables as covariates, produced essentially unchanged mediation and cluster-frailty estimates (Supplementary Table S9–S11), and analogous adjustment for hypertension and dyslipidemia — the two most prevalent comorbidities in this cohort — did not materially alter the results (Supplementary Table S12 and S13). Nonetheless, residual confounding by other prescription medications cannot be excluded, and future cohorts that capture prescription-level medication records will be needed to disentangle these effects. Eighth, the Kyotango region is a defined longevity area with relatively enriched butyrate-producing taxa compared with urban Japanese populations [17]; generalizability to other Japanese cohorts and to non-Japanese populations therefore requires confirmation.

Several implications follow. Clinically, our data suggest that joint dietary–microbial profiling may refine frailty risk stratification beyond either domain alone: identifying a community-dwelling older adult who combines a vegetable-, soy- and dairy-rich dietary pattern with a butyrate-producer-rich gut microbial profile flags an individual at markedly lower frailty risk. Mechanistically, the partial mediation through Microbe PC2 is consistent with — but does not prove — a fiber → SCFA-producer microbiota → frailty pathway in older Japanese adults, complementing similar evidence from European populations [14,16]. From a translational perspective, the findings provide hypothesis-generating support for nutritional interventions that combine fiber-rich plant foods with culturally appropriate Japanese staples. Pattern 1, the most frailty-protective pattern in our cohort, was distinctive in carrying simultaneously the highest intakes of soy, vegetables, dairy products, dietary fiber, calcium and potassium and the lowest sodium-to-potassium ratio; given that Microbe PC2 (a butyrate-producer-rich axis) lies in the mechanistic pathway from fiber to frailty, a pragmatic randomised trial in community-dwelling older Japanese adults that enriches habitual diets with soy and other legumes, vegetables and dairy — and concurrently reduces dietary salt while increasing potassium — is urgently warranted. Such a trial should test, with metagenomic and metabolomic read-outs, whether this Japanese DASH-style modification shifts the gut microbiota toward a butyrate-producer-enriched profile and improves frailty markers. In parallel, the dietary sodium-to-potassium ratio and the abundance of butyrate-producing taxa could be developed as paired nutritional and microbial biomarkers that complement clinical frailty assessment. Prospective cohorts and randomised trials will be needed to establish causal direction and quantify potential intervention effects. In conclusion, in 785 community-dwelling older Kyotango residents, dietary patterns and gut microbial clusters jointly stratified frailty across a more than nine-fold gradient, with a small but consistent statistical mediation of the fiber–frailty association via a butyrate-producer-rich microbial axis, supporting an integrated dietary–microbial approach to frailty research and prevention.

## Author contributions

Conceptulization, Y.N. and T.T.; validation and formal analysis, T.Y., H.K., K.M.; and R.I.; human cohort study management, N.O., A.A., T.K., J.N., T.K. and S.M.; writing-original draft preparation, Y.N.; writing-review and editing, K.M., R.I., and T.T.; funding acquisition, Y.N. and S.M. All authors have read and agreed to be published version of the manuscript.

## Declaration of Generative AI and AI-assisted technologies in the writing process

During the preparation of this work, the authors used Claude (Anthropic, via the Cowork interface) to assist with English language editing, paraphrasing and re-organization of text across the Abstract, Introduction, Methods, Results and Discussion, and to support computational analyses including Python scripts for principal component analysis, k-means clustering, mediation analysis with bootstrap resampling, and rendering of figures and tables. All hypothesis generation, study design, biological and clinical interpretation, scientific judgments, and final decisions on manuscript content are the authors’ own. After using this tool, the authors reviewed and edited the content as needed and take full responsibility for the content of the publication.

## Funding

This work was partly supported by MAFF Commissioned Project Study on “Project for the realization of foods and dietary habits to extend healthy life expectancy” allotted to Y.N. (Grant Number JPJ009842), and partly supported by COI-NEXT, JST Grant Number JPMJPF2210 and JPMJPF2403.

## Data statement

The raw 16S rRNA gene sequencing data generated in this study have been deposited in the NCBI Sequence Read Archive (SRA) under BioProject accession numbers PRJNA1470357 and PRJNA1440982 and are publicly available. Individual-level clinical, dietary and frailty data are not publicly available because of participant privacy considerations and the terms of the ethics approval, but de-identified data may be shared with qualified investigators upon reasonable request to the corresponding author and subject to a data-use agreement and approval by the Ethics and Conflict of Interest Committee of Kyoto Prefectural University of Medicine. All analytic code used to derive dietary patterns, gut microbial clusters, the frailty index and mediation and sensitivity analyses is available from the corresponding author upon reasonable request. A summary of the item-to-group mapping of BDHQ items to the 27 food groups, the full 32-item frailty-index deficit list and all supplementary tables and figures supporting the findings of this study are included within the article and its Supplementary Materials.

## Data availability

The data that has been used is confidential.

Data will be made available on request.

## Declaration of competing interest

YN received scholarship funds from Taiyo Kagaku Co. Ltd., Morinaga Co. Ltd., Miyarisan Pharma Co. Ltd, Morishita-Jintan Co. Ltd., Fujikko Co. Ltd., Mizkan Co. Ltd., Mykinso Co. Ltd. and KINS Co. Ltd.; a collaboration research fund from TOTO Ltd., Takeda Pharma. Co. Ltd. and Taiyo Kagaku Co., Ltd.; and received lecture fees by Takeda Pharma. Co. Ltd., Biofermin. Co. Ltd., and Miyarisan Pharma. Co. Ltd. TT received a collaboration research funds from PreMedica Inc., and lecture fees from Mochida Pharma. Co. Ltd., Yanssen Pharmaceutical K.K., EA Pharma. Co. Ltd., Mitsubishi Tanabe Pharma Corporation, Takeda Pharmaceutical Co. Ltd, AbbVie GK, Bristol-Myers Squibb, and Pfizer Japan Inc.
